# Psychosocial Predictors of Weight Loss and Quality of Life at 1 Year Post-Bariatric Surgery: A Cohort Study

**DOI:** 10.17756/jocd.2020-039

**Published:** 2020-11-23

**Authors:** Chia-Hao Damien Hsu, Dorina Kordunova, Chungwon Kim, Laura Kolbe, Allan Geliebter

**Affiliations:** Department of Psychiatry, Mt Sinai St. Luke’s, Icahn School of Medicine, New York, USA

**Keywords:** Weight loss surgery, Morbid obesity, Adjustable gastric banding, RYGB, Depression, Social anxiety

## Abstract

**Background:**

Psychosocial factors, identified in pre-bariatric surgery evaluation, may affect surgical outcomes, as well as defer surgery, making it important to identify psychosocial predictors of surgery outcomes.

**Methods:**

Baseline depressive and social anxiety symptom scores were analyzed as predictors of post-surgical weight loss (WL) and quality of life (QOL) following Roux-en-Y gastric bypass (RYGB) and adjustable gastric banding (AGB). Eighty-nine (82F, 7M) participants underwent surgery and completed depression, anxiety and QOL questionnaires 3-weeks pre-surgery and 1-year post-surgery.

**Results:**

Depressive scores and QOL scores improved post-surgery (P < 0.001), whereas social anxiety scores did not (P = 0.20). Baseline depressive (P = 0.90) and social anxiety (P = 0.20) scores did not predict % weight loss (WL) at 1 year, but higher baseline depressive (P = 0.04) and social anxiety (P = 0.005) scores predicted lower post-surgical QOL at 1 year. RYGB showed greater improvement in %WL (P < 0.001) than AGB, but no difference between the groups in QOL (P = 0.10). Improvement in QOL correlated with %WL (P < 0.001), whereas improvement in depressive scores did not correlate with %WL (P = 0.70) but did correlate with improvement in QOL (P = 0.01).

**Conclusions:**

Baseline depressive and social anxiety scores predicted QOL but not %WL. Depressive and QOL scores improved post-surgery, but social anxiety scores did not. The findings suggest that patients who present with depressive or social anxiety symptoms pre-surgery perhaps should not be deferred; however, more studies are needed to confirm this. Patients with pre-operative social anxiety symptoms may benefit from counseling.

## Background

To improve candidate selection, enhance pre-surgical counseling and identify those who may need increased post-surgical support, it is important to study potential psychological predictors of post-surgical weight loss. Psychological evaluations are standard in most bariatric surgery programs [1, 2]. Psychopathology is more common in individuals with obesity than those in the general population and more common in individuals with obesity who seek weight loss treatment than in those from community samples [1, 3, 4]. About 40% of bariatric surgery patients have at least one psychiatric diagnosis, with depressive and anxiety disorders being two of the most common [1, 4, 5]. Poorer weight loss outcomes have been shown in individuals with a history of mood disorders [4–6].

Studies of anxiety disorders as predictors of surgery outcomes are less common than mood disorders, with mixed results [1, 4], particularly studies examining social anxiety. Social anxiety disorder is characterized by fear of being embarrassed in social settings and of being judged by others based on their appearance and therefore is often present in individuals with obesity [7]. It has been shown that lifetime prevalence rates of social anxiety disorder range from 3.2% to 9.4% in bariatric surgery candidates and it was the second most prevalent disorder in a sample of nearly 2000 bariatric surgery candidates [7]. Furthermore, weight-related social anxiety may be just as severe as non-weight-related social anxiety [7]. To our knowledge, only one study looked at social anxiety in weight loss surgery (WLS), specifically biliopancreatic diversion and found a high prevalence of social anxiety both before and after surgery as compared to individuals without obesity [8]. However, in that study the authors examined two different groups, those waiting for surgery and those who had already undergone the procedure, but not within one group, pre- and post-surgery.

Identification of psychological variables that consistently predict post-surgical weight loss outcomes have been inconclusive [9, 10]. Studies on depressive symptoms have shown inconsistent findings, with depression being a positive [11, 12] or negative [13–15] predictor of weight loss, or no correlation with weight loss [10, 16, 17] following adjustable gastric banding (AGB) or Roux-en-Y gastric bypass (RYGB). This reflects the complex relationship between depression and weight change. Generalized anxiety in only one study by Hafner et al. was a significant negative predictor of weight loss at 4 years following gastric restriction surgery (mostly RYGB) [18]. In that study, pre-operative depressive symptom scores were not predictive of post-surgical weight loss at 1-year follow-up, consistent with another study [19]. However, Preiss et al. found that at 1 month and 6 months post AGB, weight loss correlated with change in depressive symptoms [20]. Less research has been done on predictors of post-surgical QOL even though poor QOL is often a concomitant of obesity [21]. In one study, QOL improved significantly in RYGB patients at 2 years post-surgery [22].

It remains unclear which factors identified in the pre-operative psychological evaluations warrant denial or deferment of surgery [2]. Although denial and deferment from surgery are uncommon, Pawlow et al. found that of 449 RYGB patients, 2.7% were considered psychologically inappropriate for surgery, and 15.8% were deferred pending psychological treatment; only 10% of those deferred, returned for surgery [23]. Thus, many patients who are deferred never receive surgery, and therefore it is essential to determine whether deferment for surgery is warranted.

RYGB results in significantly greater weight loss than AGB at 1-year follow-up [24]. Despite successful weight loss for many patients, some studies indicate that the rates of weight loss failure, defined as < 50% of excess weight loss, may be as high as 20% for all bariatric surgery patients [25, 26] and may occur after both RYGB and AGB [24].

Many studies on predictors of post-surgical weight loss have been limited by retrospective chart reviews based on pre-surgical psychological evaluations. Furthermore, the instruments used are often not standardized and vary among researchers. Due to these disparate research findings and methodologies, additional research is needed to determine which psychological factors may be predictive of weight loss following WLS, and whether they have differential effects on RYGB versus AGB, independent of weight loss. Additionally, many studies had weight loss as the only outcome measure, which is limiting, as WLS affects various areas of a patient’s life, including psychological wellbeing and QOL [1]. Since the desire to improve QOL is a common reason many patients seek WLS [27], it is important to assess improvements in QOL in patients who present with psychological symptoms pre-operatively. Furthermore, there is a paucity of research on social anxiety in relation to WLS.

The current study investigated pre-operative depressive and social anxiety symptom scores as predictors of weight loss and QOL following RYGB and AGB. A key hypothesis was that higher pre-operative depressive and social anxiety symptom scores would predict less weight loss and less improvement of QOL following WLS. Another hypothesis was that RYGB would result in greater improvement of QOL than AGB, independent of weight loss, possibly due to greater resolution of comorbidities and fewer long-term complications [24]. Lastly, it was expected that QOL, depressive and social anxiety scores would all improve post-surgery.

## Methods

### Participants and procedures

This prospective study followed a cohort from 3 weeks pre-WLS (baseline) to 1-year post-WLS. Participants (n = 89) were recruited from a bariatric surgery center at a major urban hospital in NYC during a pre-operative orientation session, and signed St. Luke’s-Roosevelt Hospital IRB approved consent forms. Participants were informed that participation would not affect their surgical process, and that their responses to the questionnaires would be kept confidential from the surgery staff. Inclusion criteria for the study were adults ages 18–75, with BMI > 40 kg/m^2^ or BMI of 35–40 kg/m^2^ with medical comorbidities, a history of suboptimal weight loss or weight loss maintenance with conventional weight loss methods, and scheduled to undergo either RYGB or AGB laparoscopic surgery with a participating surgeon. Upon completing the questionnaires, each participant was given a round-trip Manhattan Transit Authority (MTA) MetroCard or equivalent cash value to cover public transportation costs.

### Measures

Participants completed a packet of questionnaires, which included a demographic questionnaire, a depression scale (Zung Self-Rating Depression Scale: ZSDS), a social anxiety scale (Liebowitz Social Anxiety Scale-Self Report Version: LSAS-SR), and a QOL scale (Impact of Weight on Quality of Life-Lite: IWQOL-Lite) 3 weeks prior to WLS and 1 year following surgery. The follow-up assessments were coordinated with the participants’ surgical follow-up visits, and a research assistant met the participants at the surgery office to administer the questionnaires.

The Zung Self-Rating Depression Scale (ZSDS) is a validated reverse-scored instrument used to assess symptoms of depression. It is a short, self-administered survey with 20 items to rate the four common characteristics of depression: pervasive effects, physiological symptoms, psychomotor effects, and other psychological disturbances. It also includes a question on suicidal ideation. It is a valid and sensitive measure of clinical severity and has proven useful as a screening tool in clinical studies [28]. Participants are asked to mark “none or a little of the time,” “some of the time,” “good part of the time,” or “most or all of the time” in response to questionnaire items. Raw scores range from 20 to 80, which are converted to scaled scores that range from 25 to 100. Scaled scores < 50 are in the normal range, 50–59 indicate minimal mild depression, 60–69 moderate to marked depression, and > 70 severe to extreme depression.

The Liebowitz Social Anxiety Scale-Self Report version (LSAS-SR) is used to assess symptoms of social anxiety [29]. The LSAS-SR is a 24-item measure based on a 4-point Likert scale that has been validated to assess anxiety, fear, and avoidance in a variety of social situations. Scores on the LSAS-SR are divided into a performance and a social anxiety score. Scores on the performance anxiety subsection can range from 0 to 39, and scores on the social anxiety subsection can range from 0 to 33. Lower scores indicate lower levels of anxiety. The LSAS-SR has demonstrated good internal consistency (α = 0.79) and good convergent validity with the interview based LSAS (r = 0.785).

The Impact of Weight on Quality of Life-Lite (IWQOL-Lite) is a validated, 31-item, self-reported measure of obesity-specific QOL [30]. It assesses five domains: physical function, self-esteem, sexual life, public distress, and work, using 5-point Likert scales. The IWQOL-Lite has good internal consistency, good responsiveness to weight loss and weight gain [31], good test-retest reliability [32], and sensitivity to the degree of obesity [19].

Demographic data including age, gender, education, and ethnicity were obtained in a self-report questionnaire. During pre-operative and follow-up visits at the surgeon’s office, patients were weighed on a Scale-tronix medical scale (5702 Bariatric Stand-On Scale) with a capacity of 1,000 lbs (454 kg), and height was measured with a stadiometer at the baseline visit.

### Statistical analyses

First, we tested whether baseline BMI or age differed between the two surgery types by ANOVA and whether gender, race, and education differed by chi-square. No significant differences were found, and therefore these subject variables were not entered as covariates in further comparisons between the surgery groups. Additionally, none of these subject variables had a significant effect on changes in depressive or social anxiety symptom scores and therefore were not entered in the analyses of the psychosocial factors. An ANOVA was then performed to test whether or not changes in psychosocial factors varied with type of surgery, entered as a fixed factor. Paired t-tests were performed to show direction. We also ran Pearson’s correlation tests between changes in psychosocial factors and %WL, and between changes in QOL and %WL. Two-tailed α = 0.05 was the threshold for significance. To account for multiple comparisons for the t-tests, we used the Bonferroni correction with significance set at α = 0.0083 (0.05/6 for 6 comparisons). ANOVA effect size for significant findings is given as (eta squared) η^2^ where 0.02 is low, 0.13 moderate, and > 0.26 high. We used IBM SPSS Statistics (Version 26) for all analyses. A STROBE checklist was used to ensure all variables and factors were included within the manuscript.

## Results

### Sample characteristics

Eighty-nine participants (82 female, 7 male) completed the questionnaires: 65 participants (60 female, 5 male) underwent RYGB and 24 (22 female, 2 male) underwent AGB. Their characteristics are shown in table 1. Consistent with other studies, the participants who underwent bariatric surgery were mostly female [33].

### Effect across both surgeries combined

The data for RYGB and AGB were combined and analyzed. Results (Table 2) indicated that at 1 year, WLS overall resulted in decreased body weight (M = 38.7 kg, SD = 19.3, t (88) = 18.9, P < 0.001), decreased BMI (M = 14.0 kg/m^2^, SD = 6.8, t (89) = 19.3, P < 0.001), and improvements in QOL (M = −29.0, SD = 23.4, t (83) = −11.4, P < 0.001).

Depressive scores improved post-surgery (M = 5.3, SD = 10.3, t (88) = 4.9, P < 0.001), whereas social anxiety scores did not change significantly (M = 1.65, SD = 11.42, t (88) = 1.36, P = 0.20). Baseline depressive (F (1, 87) = 0.03, P = 0.86) and social anxiety (F (1, 87) = 1.6, P = 0.20) scores were not predictive of %WL. Improvement in depressive scores (r = −0.04, P = 0.70) or changes in social anxiety scores (r = −0.21, P = 0.053) did not correlate with %WL either. However, higher baseline depressive (F (1, 82) = 4.3, η^2^ = .05, r^2^ = .05, P = 0.04) and social anxiety (F (1, 82) = 8.4, η2 = 0.09, r^2^ = 0.09, *P* = 0.005) scores were predictive of lower postsurgical QOL. Furthermore, improvement in QOL correlated with %WL (r = 0.40, P < 0.001) and with improvement in depressive scores (r = −0.27, P = 0.01).

### Relationship between type of surgery and outcome variables

Outcomes were then compared between RYGB and AGB groups. Improvement in depressive scores (F (1, 87) = 0.04, η^2^ = < 0.001, P = 0.85) or changes in social anxiety scores (F (1, 87) = 0.65, η^2^ = 0.007, P = 0.42) did not vary by type of surgery. RYGB led to greater %WL compared to AGB (F (1, 87) = 109.2, η^2^ = 0.50, P = < 0.001; 35.0% ± 8.75 versus 13.4% ± 8.38) (Table 2). RYGB had an average 46.6 ± 15.3 kg weight loss, whereas AGB had a 17.3 ± 10.7 kg weight loss (F (1, 87) = 74.2, P < 0.001) (Table 2). There were no differences in the changes in QOL scores between the surgeries (F (1, 82) = 2.7, P = 0.10) (Table 2).

## Discussion

The results showed that pre-operative depressive and social anxiety did not predict weight loss at 1-year post-RYGB and AGB. Thus, our hypothesis that depressive and social anxiety scores would predict less weight loss was not supported. Following RYGB surgery, Thonney et al. similarly found that pre-surgical depressive and anxiety symptoms did not predict weight loss at 1 and 2 years post-surgery [34]. However, Jalivand et al. found that patients who exhibited depression, anxiety disorders, and/or bipolar disorder pre-operatively had lower % excess body weight loss at 6 months post-WLS (RYGB and sleeve gastrectomy) when compared to those without any psychological history, but this difference between the groups was not found at 1-year post-surgery [2].

Our results also showed that depressive scores improved post WLS, but social anxiety scores did not, thus only partially supporting our hypothesis that both would improve. The changes in depressive scores were associated with improvement in IWQOL-Lite scores, even though these changes were not correlated with %WL. Higher baseline depressive and social anxiety scores both predicted lower post-surgical QOL scores, supporting the hypothesis that baseline social anxiety and depressive symptoms would be negative predictors of QOL. Finally, consistent with other studies, the results showed that RYGB led to greater weight loss than AGB [35, 36].

Although depressive scores improved at 1 year following WLS, social anxiety symptom scores did not. We speculate that this might be related to increased social attention that many patients receive due to their weight loss following WLS, which may keep their social anxiety elevated. Social anxiety is characterized by a persistent fear of interacting or performing in social situations due to concerns of embarrassment, humiliation, or negative evaluation by others [29]. It is possible that the weight loss experienced following WLS created a unique challenge for those with symptoms of social anxiety. Furthermore, it has been estimated that 27% of surgery patients overestimate the expected postoperative weight loss, which may contribute to increased anxiety when they do not lose as much weight as anticipated [3]. However, Kalarchian et al. found that the prevalence of anxiety disorders was significantly lower at 3-years post-RYGB or AGB when compared to pre-surgery [13]. It is possible that bariatric surgery patients eventually become habituated to the newly gained attention post-surgery, and their anxiety abates.

Strengths of this study include the prospective design and the use of repeated measures within the same individuals to reduce between-subject variance. Moreover, all participants were recruited from the same hospital’s bariatric surgery center, controlling for therapeutic variability among surgical treatment centers. Furthermore, the majority of our participants fell into Black or Hispanic race categories, unusual for a study on bariatric surgery outcomes, and providing findings within these demographic groups. Limitations of the study include a relatively small sample size, uneven numbers in the surgical groups, and examining outcomes only at 1-year follow-up.

### Clinical implications

Although a number of authors discourage exclusion from WLS based on psychiatric symptomology [30, 37], this concern remains among many mental health professionals. Some recommend that the patients undergo treatment prior to surgery, thus postponing the patients’ surgery date and often deterring the surgery process altogether [38]. Others take a more absolute position and either clear such patients for surgery without treatment or recommend exclusion from surgery. The results of this study suggest a more nuanced approach, as we did not find an association between baseline depressive or social anxiety symptom scores and post-surgical weight loss. The results suggest that depressive and social anxiety symptoms may not be a contraindication for surgery.

The results also showed that social anxiety symptom scores did not improve following WLS, suggesting that those with social anxiety symptoms prior to surgery should be identified and encouraged to undergo treatment. Both psychotherapies, particularly cognitive behavioral therapy, and pharmacological treatments have been successful in managing social anxiety disorder in adults [39].

Furthermore, the study found an association between higher baseline depressive and social anxiety symptom scores and lower postoperative QOL scores. Patients identified with depressive and social anxiety symptoms at pre-surgery screening may benefit from additional psychotherapeutic support throughout the surgery process, as well as post-surgery. Although the psychosocial measures analyzed in this study did not predict weight loss, QOL is also an important outcome. which impacts overall well-being [40] and was affected by lower depressive and anxiety scores. Cognitive behavioral therapy appears to be a promising adjunct approach to help ameliorate depressive and anxiety symptoms [41], and therefore potentially improve QOL as well.

## Figures and Tables

**Table 1:** Baseline Participant Characteristics

Characteristics	RYGB		AGB		Combined	

	n = 65	Mean ± SD or %	n = 24	Mean ± SD or %	n = 89	Mean ± SD or %

**Age**		39.3±1.4		43±2.2		40.3±11.3
**Weight** (kg)		133.2±3.6		128.4±5.0		131.9±28.0
**BMI** (kg/m^2^)		47.2±7.3		46.9±9.2		47.1±7.8
**Depressive score**		46.0±9.6		48.3±9.0		46.6±9.4
**Social anxiety score**		17.1±14.4		18.8±15.5		17.5±14.6
**QOL score**		54.7±23.8		51.9±20.4		54.0±22.9
**Education**						
*Some high school*	6	9.2%	3	12.5%	9	10.1%
*High school graduate or equivalent (GED)*	13	20%	3	12.5%	16	18%
*Some college or associate degree*	25	38.5%	5	20.8%	30	33.7%
*Completed college*	21	32.3%	13	54.2%	34	38.2%
**Gender**						
*Male*	5	7.7%	2	8.3%	7	7.9%
*Female*	60	92.3%	22	91.7%	82	92.1%
**Race**						
*African American*	27	41.5%	10	41.7%	37	41.6%
*Hispanic*	25	38.5%	9	37.5%	34	38.2%
*Caucasian*	10	15.4%	4	16.7%	14	15.7%
*Asian*	1	1.5%	1	4.2%	2	2.2%
*Other*	2	3.1%	0	0%	2	2.2%

Abbreviations: RYGB, Roux-en-Y gastric bypass; AGB, adjustable gastric banding; n, sample size; SD, standard deviation; kg, kilogram; GED, General Educational Development.

No significant differences were found between types of surgery on any of the characteristics.

**Table 2:** Outcome Measures for Combined Surgeries and for Roux-en Y Gastric Bypass (RYGB) vs. Adjustable Gastric Banding (AGB)

	RYGB and AGB Combined (n = 89)			*P*-Value^a^	RYGB (n = 65)			*P*-Value^a^	AGB (n = 24)			*P*-Value^a^ (Δ2)	*P*-Value^b^ (Δ1- Δ2)
Outcomes	Pre	Post	Δ (Pre-Post)		Pre	Post	Δ1 (Pre-Post)		Pre	Post	Δ2 (Pre-Post)		
**Weight (kg)**	131.9±28.0	93.2±25.2	38.7±19.3^c^	<0.001*	133.2±29.2	86.6±22.3	46.6±15.3^c^	<0.001*	128.3±24.5	111.1±24.0	17.3±10.7^c^	<0.001*	<0.001*
**BMI**	47.5 ±8.3	33.6 ±8.4	14.0±6.8^c^	<0.001*	47.8 ±8.1	31.0 ±6.4	16.8±5.3 ^c^	<0.001*	46.8 ±9.0	40.5 ±9.4	6.2±4.0^c^	<0.001*	<0.001*
**%Weight Loss**	--	--	29.2±12.9^c^	--	--	--	35.0±8.8^c^	--	--	--	13.4±8.4^c^	--	<0.001*
**Quality of Life**	53.7±23.2	82.7±19.9	−29.0±23.4^d^ (n=84)	<0.001*	54.4±24.0	85.9±18.6	−31.5±24.8^d^(n=62)	<0.001*	51.7±20.9	73.7±21.6	−22.0±17.7^d^ (n=22)	<0.001*	0.1
**Depression Scores**	46.6±9.4	41.3±10.2	5.2±10.3^c^	<0.001*	46.0±9.6	40.8±10.3	5.2±10.6^c^	<0.001*	48.3±9.0	42.6±10.0	5.6±9.6^c^	0.008*	0.8
**Anxiety Scores**	17.5±14.6	15.9±13.6	1.6±11.4^c^	0.2	17.1±14.4	14.8±12.8	2.2±12.1^c^	0.1	18.8±15.5	18.8±15.7	0.0±9.1^c^	0.98	0.4

Abbreviations: RYGB, Roux-en-Y gastric bypass; AGB, adjustable gastric banding; n, sample size; kg, kilogram; BMI, Body Mass Index.

a Paired t-test within groups.

b ANOVA for difference in pre- and post-surgery outcomes between RYGB and AGB.

c A positive difference denotes improvement after surgery.

d A negative difference denotes improvement after surgery.

* Significant difference; *P* ≤ 0.008 after Bonferonni correction.

## Abbreviations

AGB: Adjustable Gastric Banding
GLM: General Linear Model
IWQOL-Lite: Impact of Weight on Quality of Life-Lite
LSAS-SR: Liebowitz Social Anxiety Scale-Self Report
QOL: Quality of Life
RYGB: Roux-en-Y Gastric Bypass
WL: Weight Loss
WLS: Weight Loss Surgery
ZSDS: Zung Self-Rating Depression Scale
